# CmMDb: A Versatile Database for *Cucumis melo* Microsatellite Markers and Other Horticulture Crop Research

**DOI:** 10.1371/journal.pone.0118630

**Published:** 2015-04-17

**Authors:** Bhawna, Pavan K. Chaduvula, Venkata S. Bonthala, Verma Manjusha, Ebrahimali A. Siddiq, Ananda K. Polumetla, Gajula M. N. V. Prasad

**Affiliations:** 1 National Bureau of Plant Genetic Resources, Genomics resources div., New Delhi-12, India; 2 National Research Center on Plant Biotechnology, Pusa campus, New Delhi-110012, India; 3 Institute of Biotechnology, PJTSAU, Rajendra Nagar, Hyderabad-500030, India; National Institute of Plant Genome Research, INDIA

## Abstract

*Cucumis melo* L. that belongs to Cucurbitaceae family ranks among one of the highest valued horticulture crops being cultivated across the globe. Besides its economical and medicinal importance, *Cucumis melo* L. is a valuable resource and model system for the evolutionary studies of cucurbit family. However, very limited numbers of molecular markers were reported for *Cucumis melo* L. so far that limits the pace of functional genomic research in melon and other similar horticulture crops. We developed the first whole genome based microsatellite DNA marker database of *Cucumis melo* L. and comprehensive web resource that aids in variety identification and physical mapping of Cucurbitaceae family. The *Cucumis melo* L. microsatellite database (CmMDb: http://65.181.125.102/cmmdb2/index.html) encompasses 39,072 SSR markers along with its motif repeat, motif length, motif sequence, marker ID, motif type and chromosomal locations. The database is featured with novel automated primer designing facility to meet the needs of wet lab researchers. CmMDb is a freely available web resource that facilitates the researchers to select the most appropriate markers for marker-assisted selection in melons and to improve breeding strategies.

## Introduction

Melon (*Cucumis melo* L.) (2n = 2x = 24) is an important eudicot diploid horticultural crop with an estimated haploid genome size of 454 Mb. The melon belongs to the *Cucurbitaceae* family, which also contains other important crops such as cucumber, watermelon and pumpkin. It is cultivated worldwide, mainly in temperate, subtropical and tropical regions [1]. Melon is well known for its specific biological, medicinal and economic significance. Melon is amongst important fleshy fruits used for fresh consumption [2]. Melon displays high variability in its physical, biochemical and phenotypic characteristics and also acts a stimulator for precursors of modern genetics [3]. According to recent statistics available from the USDA reports of 2009, melon production has reached 31,053,716 tons worldwide with an increase of just 0.3% compared to its previous year’s production [4]. The major melon producing countries are China with the share of 52% of total global production followed by USA, Spain, Turkey and Iran respectively in the order of production [4].

Various molecular markers have been developed and extensively used to monitor genomic divergence in and among species for breeding and genetic analysis studies [5]. In recent years, microsatellite markers gained much importance in various genetics and molecular studies because of their high co-dominant inheritance, reproducibility, multi-allelic variation and their abundance in the genome. These microsatellite markers have been employed in various applications of structural, functional and comparative genomics (syntenic and phylogenetic study), variety identification, marker-assisted selection and construction of high density genome maps [6–13]. Despite of melon's economic and scientific importance, there exists only a limited numbers of microsatellite markers and resources. There were numerous attempts to develop SSR markers and resources for various important crops like bottle gourd [14], watermelon [15], cucumber [16], melon [17], foxtail millet [18–20], tomato [21], barely [22], potato [23], sugarcane [24], capsicum [25], and eggplant [26].

A number of genetic and molecular resources were developed over the last few years in the field of functional genomics, including the development of physical maps [27, 28], ESTs [29, 30] and microarrays [31, 32]. However, a very limited numbers of molecular markers were developed for melon despite its various scientific and economic importance [33]. Therefore, it is necessary to develop a large scale catalogue of microsatellite DNA marker by taking advantage of the availability of whole genome sequence of melon [34]. Considering the employability of whole genome sequence-based microsatellite markers, we made an attempt to identify and develop microsatellite markers from melon genome and subsequently develop a relational database to provide unrestricted access to plant breeders and researchers.

## Materials and Methods

### Database processing pipeline

**Fig 1:**
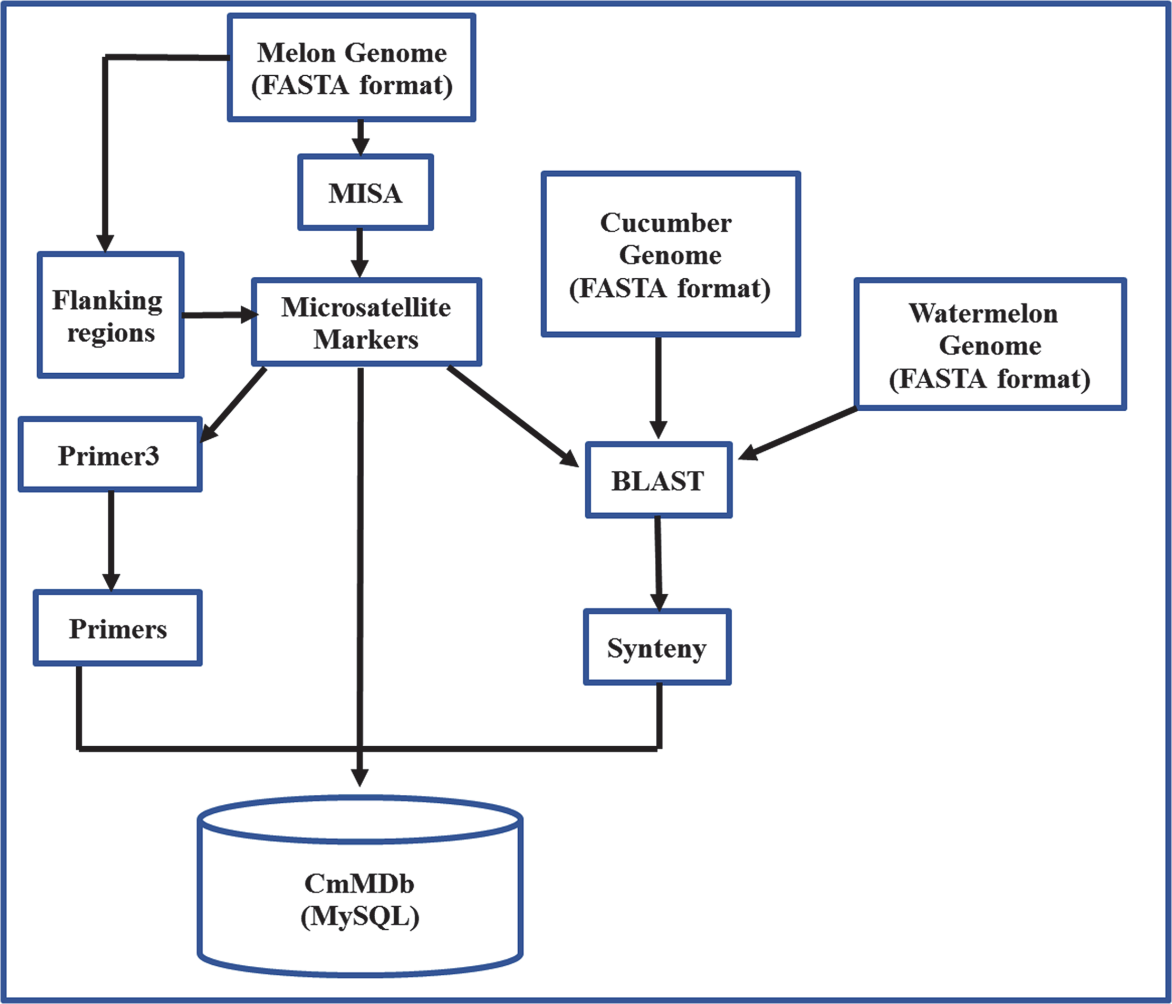
Database processing pipeline.

The draft of whole genome sequences of *Cucumis melo* L. [34] was downloaded in FASTA format from the melonomics website [35] and fed to Micro Satellite (MISA) identification tool [36] to identify the microsatellite markers with the following search criteria: 6 repeat units used for dinucleotide repeats (DNRs), 5 repeat units used for rest all nucleotides including trinucleotide repeats (TNRs), tetranucleotide repeats (TeNRs), pentanucleotide repeats (PNRs) and hexanucleotide repeats (HNRs). The primer pairs were designed from either side of flanking regions of the identified microsatellite markers using integrated Perl 5 interface module of MISA-Primer3 software [36]. The identified microsatellite markers were mapped by using BLAST software [37] with default E-value onto the melon's chromosomes. The flanking sequences of microsatellite markers of melon were then submitted to BLAST [37] against genome sequences of cucumber and watermelon to study the marker-assisted syntenic relationships among the chromosomes of *C*. *melo*, cucumber and watermelon. A cutoff E-value of 1e-05 was considered significant for the BLAST analysis. Finally, the identified microsatellites with their corresponding primer pairs and the syntenic information were stored in MySQL database [38]. Fig 1 represents the entire processing pipeline of CmMDb.

### Database architecture

**Fig 2:**
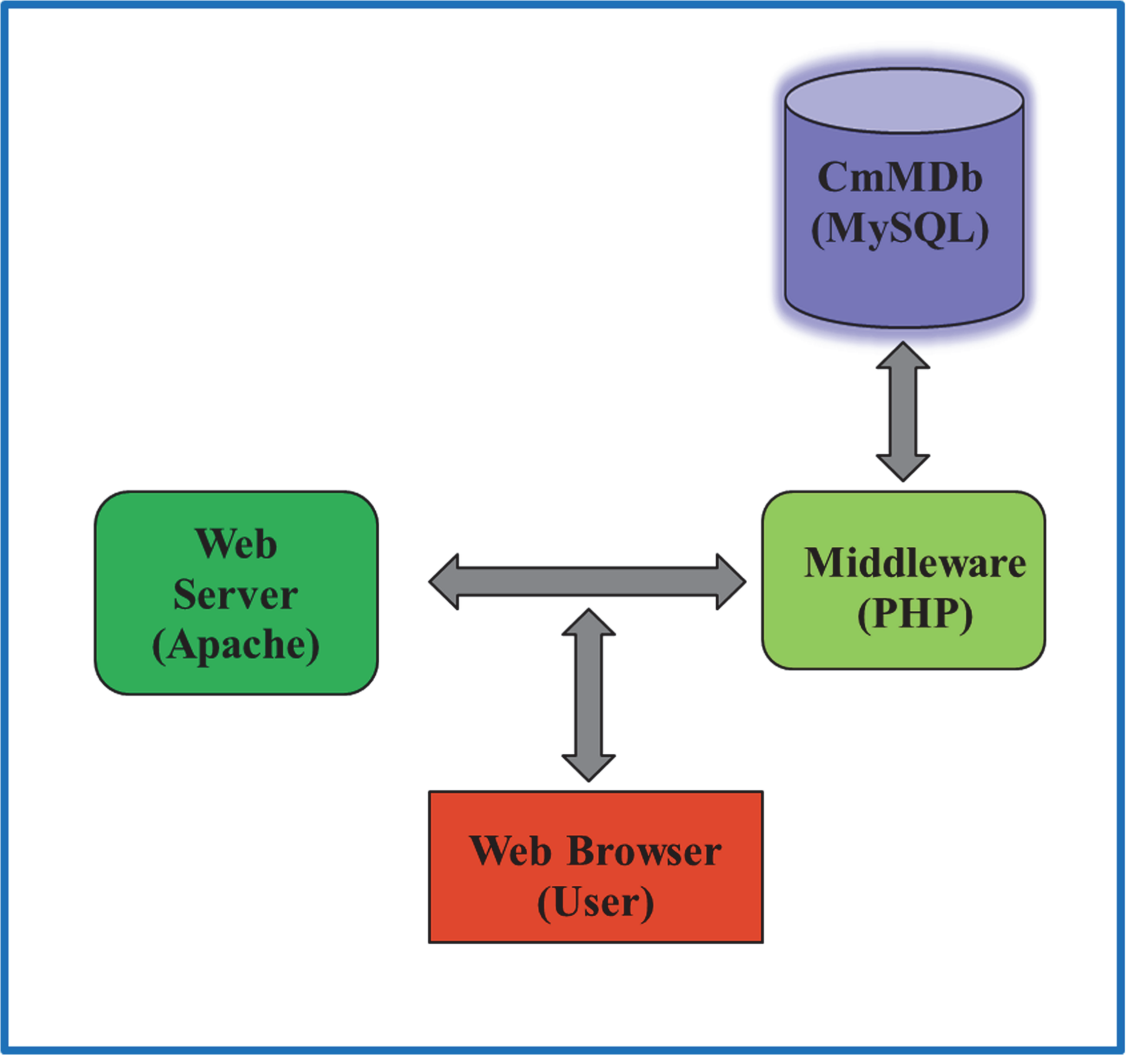
Three-level schema architecture of CmMDb.

**Fig 3:**
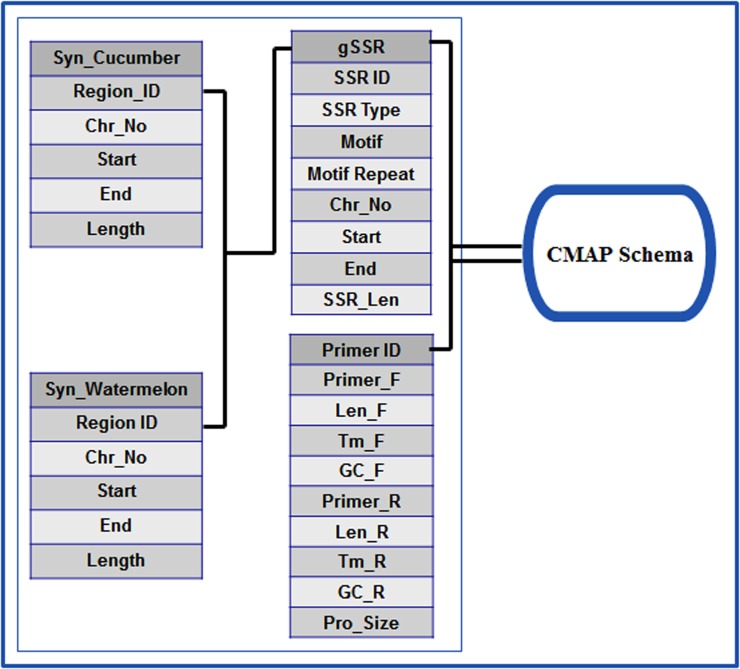
CmMDb Database schema.

*Cucumis melo* Microsatellites Database (CmMDb) is an online relational and interactive database. The database was designed based on “Three-Level Schema Architecture” as represented in Fig 2 and was developed by using MySQL 5.0 [38] to store all the information associated with microsatellite markers. The CMap [39] schema has been integrated with CmMDb to facilitate interactive visualization of physical map and for comparative mapping (syntenic information) of the marker data with genomes of cucumber and watermelon. The schema of CmMDb is shown in Fig 3.

### User interface

The user-friendly interface for CmMDb was developed using open-source server side scripting language, PHP 5.4 and HTML to query and retrieve the results from the database as per user requirements. The database can then be searched either by providing user specific input (gSSR tab) or by browsing the interactive physical map (Map tab). The 'gSSR' tab provides different search parameters to search the entire CmMDb or to choose the desired microsatellite markers based on motif repeat and motif length, motif sequence, marker ID, chromosome number and motif type.

**Fig 4:**
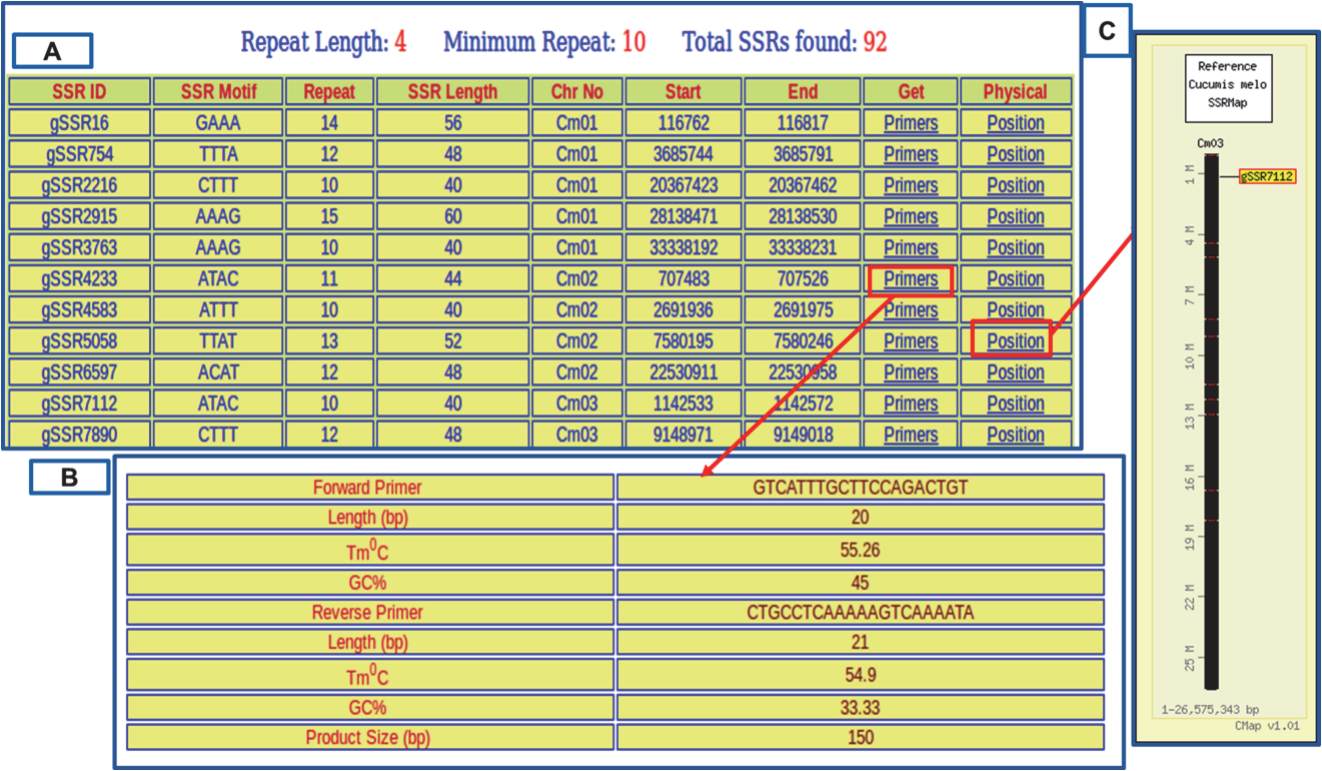
CMap's User Interface presenting a marker details along with its physical position.

**Fig 5:**
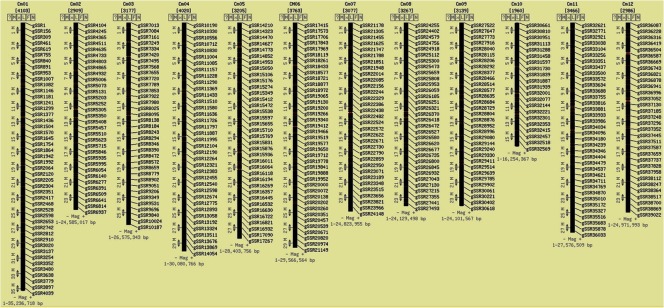
Physical map of the *Cucumis melo* generated by the CMap interface.

**Fig 6:**
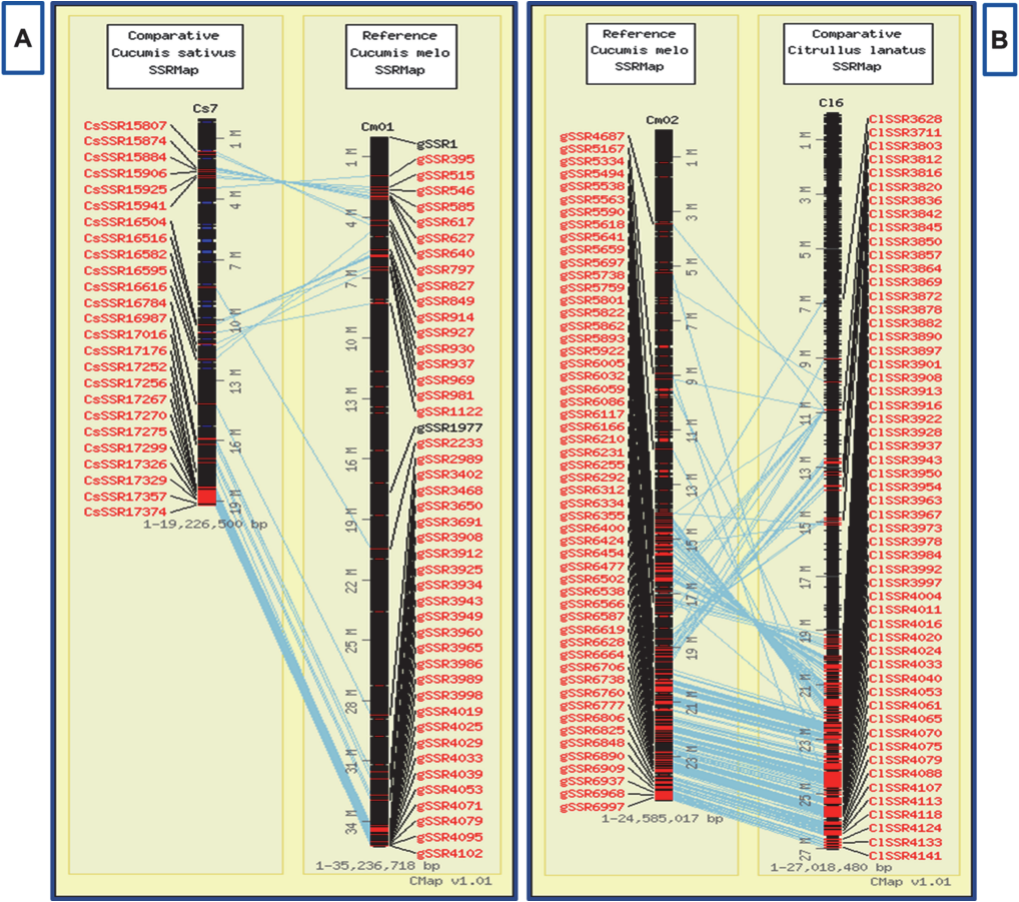
A segment of comparative map displayed through CMap interface showing the syntenic correspondences (in blue lines) between (A) *Cucumis melo* chromosome 1 and *Cucumis sativus* chromosome 7, (B) *Cucumis melo* chromosome 2 and *Citrullus lanatus* chromosome 16.

The result of the respective query will be presented in a tabular format with marker ID, motif sequence, motif repeat, chromosomal location, start and end positions along with hyperlinks to corresponding primer information and physical map (Fig 4A). The hyperlink provided for ‘Primers’ under 'Get' column redirects to the primer data page that lists information about the corresponding primer which include forward and reverse primer sequences, respective melting temperatures (Tm), lengths and expected product size (Fig 4B). The 'Position' hyperlink under 'Physical' column redirects to CMap interface [39] where the physical locations of the markers in the melon genome can be viewed interactively (Fig 4C). The 'Map' tab gives CMap [39] based interactive physical map of all the microsatellite markers on the twelve chromosomes of the melon genome (Fig 5). This interactive map allows the user to study the syntenic relationships among any chromosome or all the chromosomes of the melon genome and cucumber and/or watermelon chromosomes by drawing interactive comparative maps (Fig 6).

Finally, the complete marker data of CmMDb can be downloaded from the ‘Download’ section of the CmMDb. In addition to the self-explanatory usage of the interface, a robust tutorial has also been built for better user understanding.

## Results and Discussion

The available whole genome (375 Mb) sequences of melon [34] was searched for microsatellites, and a total of 39,702 microsatellite markers comprising different kinds of desirable motif-repeats (from DNRs to HNRs) were identified with the average frequency of about 123 microsatellites per mega base sequences (Mb). The DNR type (61.13%) was found to be dominant, followed by TNR type (30.66%), TeNR type (5.59%), PNR type (1.83%) and the HNR type (0.77%). Chromosome 1 contains the highest number of markers (4103) while chromosome 10 exhibits a minimum number of microsatellite markers (1960) (Table 1). Among DNRs, AT/TA motifs (71.91%) were more frequent, followed by AG/GA (10.19%), CT/TC (10.08%), AC/CA (4%), TG/GT (3.76%) and GC/CG (0.04%). Among the TNRs, AAT/TAA (21.29%) motifs were most abundant followed by ATT/TTA (19.11%), AAG/GAA (10.87%) and TTC/CTT (10.49%), whereas CCG/GCC (0.47%) followed by TGG/GGT (0.77%) motifs were less abundant. Based on the length of the repeat motifs, a total of 1,835 (4.62%) microsatellite markers was classified as long and hypervariable class I (≥20 bp) types, a total of 4,684 (11.79%) microsatellites as variable class II (12–19 bp) types and remaining 32,553 (81.99%) microsatellites as variable class III (5–11 bp) types. All data related to distribution and frequencies of markers are based on SSR class and predominant motif present in DNR and TNR. microsatellites as shown in S1 Table, its parts S1, S1.1 and S1.2, respectively.

**Table 1:** Distribution of different types of microsatellites present in each chromomsome, their marker percentage and density in melon genome.

Chr	DNR	TNR	TeNR	PNR	HNR	Total	Chr Size (bp)	Markers %	Density (Markers/Mb)
Cm01	2531	1252	222	68	30	4103	35236718	10.50	116.46
Cm02	1754	923	151	56	25	2909	24585017	7.45	118.35
Cm03	1884	1029	171	64	29	3177	26575343	8.13	119.57
Cm04	2504	1180	227	81	28	4020	30080766	10.29	133.64
Cm05	1976	958	191	65	15	3205	28403756	8.20	112.85
Cm06	2316	1137	225	54	31	3763	29566564	9.63	127.30
Cm07	1864	960	171	62	20	3077	24823955	7.88	123.97
Cm08	1951	1021	209	62	24	3267	24129498	8.36	135.45
Cm09	1944	926	178	67	24	3139	24101567	8.03	130.25
Cm10	1198	620	102	24	16	1960	16254367	5.02	120.62
Cm11	2114	1059	194	61	38	3466	27576509	8.87	125.72
Cm12	1852	915	145	52	22	2986	24971993	7.64	119.58
**Total**	**23888**	**11980**	**2186**	**716**	**302**	**39072**			

To study the marker-assisted syntenic relationships between melon, cucumber and watermelon chromosomes, the physically mapped melon microsatellite markers were submitted to BLAST against genomes of cucumber and watermelon with e-value of 1e-05. The comparative genome mapping showed 44.46% (17,375) of the melon markers have sequence-based orthology and syntenic relationships with cucumber while 18.67% (7,298) melon markers have sequence-based orthology and syntenic relationships with watermelon genomes, respectively. The 4^th^ chromosome of melon has shown maximum synteny (1,632 markers) with cucumber followed by 6^th^ chromosome of melon (1,442 markers), whereas the 3^rd^ chromosome of melon shown maximum synteny (463 markers) with watermelon followed by 1^st^ chromosome of melon (447 markers). All the comparison among the chromosomal wise distribution of microsatellite markers between melon with that of watermelon and cucumber is presented in S2 and S3 Tables, respectively.

CmMDb serves as a repository of DNA markers and can be employed as a tool in marker-assisted breeding programme of melon's improvement, genetic diversity and syntenic studies between the Cucurbitaceae members. CmMDb can be of much support in browsing or searching for a particular marker on the genome interactively and also to visualize each marker on the chromosome by using CMap interface [39]. The relative density of the melon whole genome reported in the study is 123markers/Mb, while the other closest model plant genome is Arabidopsis with 157 Mb. Whereas in comparison to other crops such as cucumber (367 Mb), rice (370–490 Mb), popular (485 Mb), grape (487 Mb), sorghum (818 Mb), soybean (1115 Mb), maize (2365 Mb), wheat (1000 Mb) and pigeon pea (833 Mb) [36] reported with higher number of markers.

CmMDb is of great use to melon breeders in molecular breeding. This database contains a number of options which facilitate easy search and retrieval of a specific marker on a chromosome or on a specific portion of the chromosome and corresponding primer pairs along with their physical map (CMap interface) [39]. This database contains sequence-based orthology and syntenic relationships for each microsatellite markers with cucumber and watermelon and the same can be visualized using a CMap interface. CmMDb is useful to study the marker-assisted syntenic studies between the melon's chromosomes and chromosomes of cucumber and watermelon. CmMDb can be resourceful for mining information in order to design experiments in directions of interpreting novel roles and functions of microsatellites.

## Conclusion

CmMDb contains the information related to genomic microsatellite markers, with unrestricted public access. Based on available genome sequence data, genomic SSR markers were examine in the present study. It identified a total of 39,072 microsatellite markers in available whole genome sequences of melon. Motif frequencies decreased as there was increased in motif length. as for DNR type it was 61.13% which was found to be dominant, followed by TNR, TeNR, PNR and HNR as 30.66%, 5.59%, 1.83% and 0.77% respectively. The highest number of markers is present in chromosome 1 while a minimum number of microsatellite markers is present in chromosome 10. The identified markers can be acclimated to tag a specific biological trait of melon such as biotic or abiotic stress resistant traits (QTLs) to further develop high yielding and resistant melon varieties. Furthermore, the researchers who have developed SSR markers in *Cucumis melo* with the purpose of availing the *Cucumis melo* amendment are invited to submit their marker data to CmMDb.

## Supporting Information

S1 Table S1. Distribution and frequency of different classes of marker in *Cucumis melo* genome. **S1.1.** Distribution and frequency of different kinds of motifs based on motif repeats in microsatellite markers of *Cucumis melo*. **S1.2.** Statistics of predominent motifs found in DNR and TNR microsatellite markers of *Cucumis melo*.
(XLSX)

S2 Table Showing chromosomal wise comparision of microsatellite markers among melon and cucumber. (XLSX)

S3 Table Showing chromosomal wise comparision of microsatellite markers among melon and watermelon. (XLSX)

## References

[1] Arumuganathan K, Earle ED. Nuclear DNA content of some important plant species. Plant Mol Biol Rep. 1991; 9: 208–218. 10.1007/BF02672069

[2] Nunez-Palenius HG, Gomez-Lim M, Ochoa-Alejo N, Grumet R, Lester G, Cantliffe DJ. Melon fruits: genetic diversity, physiology, and biotechnology features. Crit Rev Biotechnol. 2008; 28:13–55. 10.1080/07388550801891111 18322855

[3] Miccolis V, Saltveit ME. Morphological and physiological changes during fruit growth and maturation of seven melon cultivars. J Am Soc Hort Sci. 1991; 116:1025–1029.

[4] FreshPlaza. Available: http://www.freshplaza.com/. Accessed 2014 Oct 23.

[5] Ferreira ME, Grattapaglia D. Introduction to the use of markers molecular genetic analysis 1998; 3rd ed Brasilia: CENARGEN Embrapa, pp.220.

[6] Delseny M, Laroche M, Penon P. Detection of sequences with Z-DNA forming potential in higher plants. Biochem Biophys Res Commun. 1983; 116:113–20. 10.1016/0006-291X(83)90388-1 6639651

[7] Tautz D, Renz M. Simple sequences are ubiquitous repetitive components of eukaryotic genomes. Nucleic Acids Res. 1984; 12:4127–4138. 10.1093/nar/12.10.4127 6328411 PMC318821

[8] Varshney RK, Graver A, Sorrells ME. Genic microsatellite markers in plants features and applications. Trends Biotechnol.2005;23 48–55. 10.1016/j.tibtech.2004.11.005 15629858

[9] Parida SK, Rajkumar KA, Dalal V, Singh NK, Mohapatra T. Unigene derived microsatellite markers for the cereal genomes. Theor Appl Genet. 2006; 112:808–17. 10.1007/s00122-005-0182-1 16429310

[10] Parida SK, Kalia SK, Sunita K. Informative genomic microsatellite markers for efficient genotyping applications in sugarcane. Theor Appl Genet. 2009; 118:327–38. 10.1007/s00122-008-0902-4 18946655

[11] Parida SK, Yadav DK, Mohapatra T. Microsatellites in Brassica unigenes relative abundance, marker design and use in comparative physical mapping and genome analysis. Genome. 2010; 53:55–67. 10.1139/g09-084 20130749

[12] Lin HS, Chiang CY, Chang SB, Kuoh CS. Development of simple sequence repeats (SSR) markers in Setaria italica (Poaceae) and cross-amplification in related species. Int J Mol Sci. 2011; 127:835–45. 10.3390/ijms12117835 PMC3233442 22174636

[13] Gupta S, Kumari K, Sahu PP, Vidapu S, Prasad M Sequence based novel genomic microsatellite markers for robust genotyping purposes in foxtail millet [*Setaria italica* (L) P Beauv]. Plant Cell Rep. 2012; 31:323–37. 10.1007/s00299-011-1168-x 21993813

[14] Bhawna, Abdin MZ, Arya L, Verma M (2014) Transferability of cucumber microsatellite markers used for phylogenetic analysis and population structure study in bottle gourd (*Lagenaria siceraria* (Mol.) Standl.). Appl Biochem Biotechnol:[Epub ahead of print]. 10.1007/s12010-014-1395-z 25471016

[15] Ren Y, McGregor C, Zhang Y, Gong G, Zhang H, Guo S. et al An integrated genetic map based on four mapping populations and quantitative trait loci associated with economically important traits in watermelon (*Citrullus lanatus*). BMC Plant Biol. 2014; 14:33 10.1186/1471-2229-14-33 24443961 PMC3898567

[16] Aurora D, Mohamed F, Gelsomina F, Peio Z, José B, Zhanjun F, et al A consensus linkage map for molecular markers and Quantitative Trait Loci associated with economically important traits in melon (*Cucumis melo* L.). BMC Plant Biol. 2011; 11: 111 10.1186/1471-2229-11-111 21797998 PMC3163537

[17] Pablo FC, Douglas AS, Luming Y, Philipp WS, Timothy TH, Chinnappa DK. et al Genome-wide characterization of simple sequence repeats in cucumber (*Cucumis sativus* L.). BMC Genomics. 2010; 11:569 10.1186/1471-2164-11-569 20950470 PMC3091718

[18] Garima P, Gopal M, Kajal K, Sarika G, Swarup KP, Debasis C. et al Genome-Wide Development and Use of Microsatellite Markers for Large-Scale Genotyping Applications in Foxtail Millet [*Setaria italica* (L.)]. DNA Res. 2013; 20:197–207. 10.1093/dnares/dst002 23382459 PMC3628449

[19] Kajal K, Mehanathan M, Gopal M, Sarika G, Alagesan S, Swarup KP, et al Development of eSSR-Markers in Setaria italica and Their Applicability in Studying Genetic Diversity, Cross-Transferability and Comparative Mapping in Millet and Non-Millet Species. PLoS ONE.2013; 8:e67742 10.1371/journal.pone.0067742 23805325 PMC3689721

[20] Venkata SB, Mehanathan M, Gopal M, Manoj P. FmMDb: A Versatile Database of Foxtail Millet Markers for Millets and Bioenergy Grasses Research. PLoS ONE. 2013;8:e71418 10.1371/journal.pone.0071418 23951158 PMC3741111

[21] Suresh BV, Roy R, Sahu K, Misra G, Chattopadhyay D. Tomato Genomic Resources Database: An Integrated Repository of Useful Tomato Genomic Information for Basic and Applied Research. PLoS ONE. 2014; 9: e86387 10.1371/journal.pone.0086387 24466070 PMC3897720

[22] Karakousis A, Barr AR, Chalmers KJ, Ablett GA, Holton TA, Henry RJ. et al Potential of SSR markers for plant breeding and variety identification in Australian barley germplasm. Aust J of Agr Res. 2003; 54:1197–1210. 10.1071/AR02178

[23] Kawchuk LM, Martin RF, Mcpherson J. Resistance in transgenic potato expressing the Potato leafroll virus coat protein gene. Mol Plant Microbe Interactions. 1990; 3:301–307

[24] Manigbas NL, Villegas LC. Microsatellite Markers in Hybridity tests to identify true hybrids of sugarcane. Philipp J Crop Sci. 2004; 29:23–32.

[25] Shirasawa K, Ishii K, Kim C, Ban T, Suzuki M, Ito T. et al Development of Capsicum EST-SSR markers for species identification and in silico mapping onto the tomato genome sequence. Mol Breeding. 2013;31:101–110. 10.1007/s11032-012-9774-z 23316112 PMC3538017

[26] Stagel A, Portis E, Toppino L, Rotino GL, Lanteri S. Gene-based microsatellite development for mapping and phylogeny studies in eggplant. BMC Genomics. 2008; 9:357–370. 10.1186/1471-2164-9-357 18667065 PMC2527019

[27] González VM, Garcia-Mas J, Arús P, Puigdomènech P. Generation of a BAC-based physical map of the melon genome. BMC Genomics. 2010; 28:339 10.1186/1471-2164-11-339 PMC2894041 20509895

[28] Harel-Beja R, Tzuri G, Portnoy V, Lotan-Pompan M, Lev S, Cohen S, et al A genetic map of melon highly enriched with fruit quality QTLs and EST markers, including sugar and carotenoid metabolism genes. Theor Appl Genet. 2010;121:511–33. 10.1007/s00122-010-1327-4 20401460

[29] Christian C, Tarek J, Yi Z, Delphine J, Mingyun H, Veronica T. et al Analysis of expressed sequence tags generated from full-length enriched cDNA libraries of melon. BMC Genomics. 2011; 12:252 10.1186/1471-2164-12-252 21599934 PMC3118787

[30] Daniel GI, José B, Cristina R, Mireia GT, Belén P, Verónica T, et al MELOGEN: an EST database for melon functional genomics. BMC Genomics. 2007; 8:306 10.1186/1471-2164-8-306 17767721 PMC2034596

[31] Ron O, Ravit E, Rotem HB, Galil T, Vitaly P, Yoseph B. et al High-throughput marker discovery in melon using a self-designed oligo microarray. BMC Genomics. 2010; 11:269 10.1186/1471-2164-11-269 20426811 PMC2874814

[32] Albert MC, Joaquin C, Josep VB, Santiago MG, José B, Daniel GI, et al An oligo-based microarray offers novel transcriptomic approaches for the analysis of pathogen resistance and fruit quality traits in melon (*Cucumis melo* L.). BMC Genomics. 2009; 10:467 10.1186/1471-2164-10-467 19821986 PMC2767371

[33] Patricia SR, Tulio CDLL, Rodrigo LT, Gláucia SCB, José AB, Márcio EF. Development of microsatellite markers from an enriched genomic library for genetic analysis of melon (*Cucumis melo* L.). BMC Plant Biology. 2004;4:9 10.1186/1471-2229-4-9 15149552 PMC419974

[34] Garcia-Mas J, Benjak A, Sanseverino W, Bourgeois M, Mir G, Gonz´alez VM, et al The genome of melon (*Cucumis melo* L.). Proc Natl Acad Sci. 2012, 109:11872–11877. 10.1073/pnas.1205415109 22753475 PMC3406823

[35] Melonomics. Available: http://melonomics.net. Accessed 2014 Sep 12.

[36] MicroSatellite analysis tool. Available: http://pgrc:ipk-gatersleben:de/misa. Accessed 2014 Sep 14.

[37] Altschul SF, Gish W, Miller W, Myers EW, Lipman DJ. Basic local alignment search tool. J Mol Biol. 1990; 215:403–410. 2231712 10.1016/S0022-2836(05)80360-2

[38] MySQL. Available: http://www.mysql.com. Accessed 2014 Jul 3.

[39] Youens-Clark K, Faga B, Yap IV, Stein L, Ware D. CMap 1.01: a comparative mapping application for the Internet. Bioinformatics.2009; 25: 3040–3042. 10.1093/bioinformatics/btp458 19648141 PMC2773250

